# Sociodemographic and clinical characteristics of persons who experienced spontaneous hepatitis C viral clearance

**DOI:** 10.1186/s12879-019-4223-9

**Published:** 2019-07-15

**Authors:** Mabel Michille Kimble, Marjan Javanbakht, Kara W. Chew, Chrysovalantis Stafylis, Di He, Samantha Ramirez, Yeonsoo Baik, Sammy Saab, Jeffrey D. Klausner

**Affiliations:** 1 Department of Medicine, Division of Infectious Diseases, University of California Los Angeles, 10920 Wilshire Blvd. Suite 350 Room 40, Los Angeles, CA 90024 USA; 2 Department of Epidemiology, Fielding School of Public Health, University of California Los Angeles, Los Angeles, USA; 3 Department of Medicine and Surgery, David Geffen School of Medicine, University of California Los Angeles, Los Angeles, USA

**Keywords:** Hepatitis C, Epidemiology, Spontaneous clearance

## Abstract

**Background:**

In the United States Hepatitis C virus (HCV) viral clearance is estimated to range between 20 and 30%. The objective of this study was to estimate the frequency of HCV clearance and identify correlates of viral clearance among patients newly identified as HCV antibody positive in a large urban health system in Los Angeles, California.

**Methods:**

We identified patients between November 2015 and September 2017 as part of a newly implemented HCV screening and linkage-to-care program at University of California Los Angeles (UCLA) Health System. All patients were eligible for screening, though there were additional efforts to screen patients born between 1945 and 1965. We reviewed Medical records to categorize anti-HCV antibody positive patients as having spontaneously cleared HCV infection (HCV RNA not detected) or not (HCV RNA detected). We excluded those with a prior history of anti-HCV positivity or history of HCV treatment. We compared differences between those with and without detectable HCV RNA using chi-square test, Fisher’s exact test, and t-test as appropriate. We assessed factors associated with HCV clearance using logistic regression analysis.

**Results:**

Among the 320 patients included in this study, 56% were male. Baby boomers (52–72 years of age) comprised the single largest age group (62%). We found spontaneous HCV clearance in 58% (*n* = 185). HCV viral clearance was slightly higher among women as compared to men (63% vs. 53%; *p* value = 0.07) and varied by race/ethnicity: clearance among Blacks/African Americans was 37% vs. 58% among whites (p value = 0.02). After adjusting for age, race/ethnicity, and sex we found that those diagnosed with chronic kidney disease had a tendency of decreased HCV viral clearance (adjusted OR = 0.34; 95% CI 0.14–1.03).

**Conclusion:**

Of those patients newly identified as anti-HCV positive, 58% had cleared HCV virus, while the rest showed evidence of active infection. In addition, we found that clearance varied by race/ethnicity and clinical characteristics.

## Background

In the United States the proportion of those with spontaneous hepatitis C virus (HCV) clearance varies between studies. Historically it’s been estimated that clearance occurs in 15–20% of patients infected with HCV [1]. Recent data suggest that spontaneous clearance of HCV infection in the absence of treatment is higher than previously estimated [1–3]. In one of the first studies to estimate spontaneous HCV clearance –defined on the basis of HCV RNA assessments within 24 months of diagnosis – the frequency of clearance was 26% [4].

Why certain groups clear HCV infection without antiviral treatment remains unclear [5–8]. Studies suggest host innate immune system or genetic factors may play a role [9–11]. Factors such as sex, race/ethnicity, young age, HLA type, IL28B genotype, HIV-infection status, and chronic hepatitis B infection are known to affect clearance [2, 4, 10, 12–21]. However, most studies on spontaneous HCV viral clearance are limited by small sample size, heterogeneous definitions of cases, and study inclusion criteria, including study populations that are limited to high-risk groups such as illicit substance users and men who have sex with men [4, 7, 12, 13]. Little is known about screening populations, in particular baby boomers, a birth cohort known to have increased risk of HCV infection [22–25]. Our aim was to identify the frequency of and factors associated with spontaneous HCV clearance among patients participating in a hepatitis C screening program at a large urban health system in Los Angeles, California.

## Methods

### Study population

Patients seeking primary care services at UCLA Health were screened for HCV infection between November 2015 and September 2017. As per the US preventive task force recommendations for HCV screening, testing was initiated with the HCV antibody test (anti HCV Ab) and was targeted to high risk groups, as well as one-time testing of adults born between 1945 and 1965 regardless (regardless of their HCV risk factors) [26]. The program has been previously described [27]. Briefly, a structural intervention using electronic medical record prompts was implemented to promote HCV screening among patients seeking care at UCLA health. The electronic medical record (EMR) prompts were specifically developed to not only capture high risk groups for HCV infection, who had never tested for HCV, but also to target screening towards “baby boomers” (i.e., those born between 1945 and 1965) without any prior history of HCV testing.

In this study, we reviewed the medical records of patients who were HCV antibody positive during the screening period (“*newly identified cases*”). We also conducted interviews to confirm history of treatment or testing history for HCV. Patients who reported that they were under treatment or had received treatment for HCV in the past were excluded from this study. Patients who reported positive HCV antibody or RNA test results before the screening period were also excluded.

### Measures

HCV antibody testing was conducted by the UCLA microbiology laboratory, using standard protocols for the ADVIA Centaur XP System (Siemens Medical Solutions USA, Inc., Malven, PA). The ADVIA Centaur HCV assay is an in vitro diagnostic immunoassay for the qualitative determination of immunoglobulin G (IgG) antibodies to hepatitis C virus in human serum and plasma using the ADVIA Centaur® System. The assay was used in conjunction with HCV RNA testing to determine HCV infection status. Specifically, HCV antibody reactive tests were tested for HCV RNA viral load using the COBAS® Ampliprep/ COBAS® TaqMan® HCV test (Qualitative and Quantitative, v2.0; Pleasanton, California). We collected patient demographic and clinical characteristics including age, sex, race/ethnicity, and laboratory values including liver function tests (aspartate aminotransferase (AST), alanine aminotransferase (ALT), platelet count, bilirubin levels) and, human immunodeficiency virus (HIV) infection status. In cases where these laboratory tests were not conducted on the same day as the HCV testing, we matched the laboratory testing data to the HCV testing data such that only testing data within 3 months of HCV antibody screening was included. We used ICD-10 codes (ICD-10 code for CKD: ICD-10-CM: N18* and ICD-10 code for DM: E11* and E10*) to determine whether patients were diagnosed with chronic kidney disease (CKD) or diabetes mellitus. These clinical markers were included in the study given the scientific literature indicating the association between CKD, diabetes, and chronic HCV infection [28–31].

### Statistical analysis

We defined HCV clearance as evidence of HCV Ab reactivity and HCV RNA non-detectable test results in the absence of treatment. We examined differences between those who cleared their HCV infection and those who remained chronically infected using chi-square test, Fisher’s exact test, and t-tests. We used logistic regression analysis to examine factors associated with HCV clearance including sociodemographic and clinical characteristics. We conducted all analyses using SAS 9.4 (NC, USA).

## Human subjects

The UCLA Institutional Review Board (IRB) approved the study activities (IRB# 15–001226).

## Results

### Study population characteristics

**Fig. 1:**
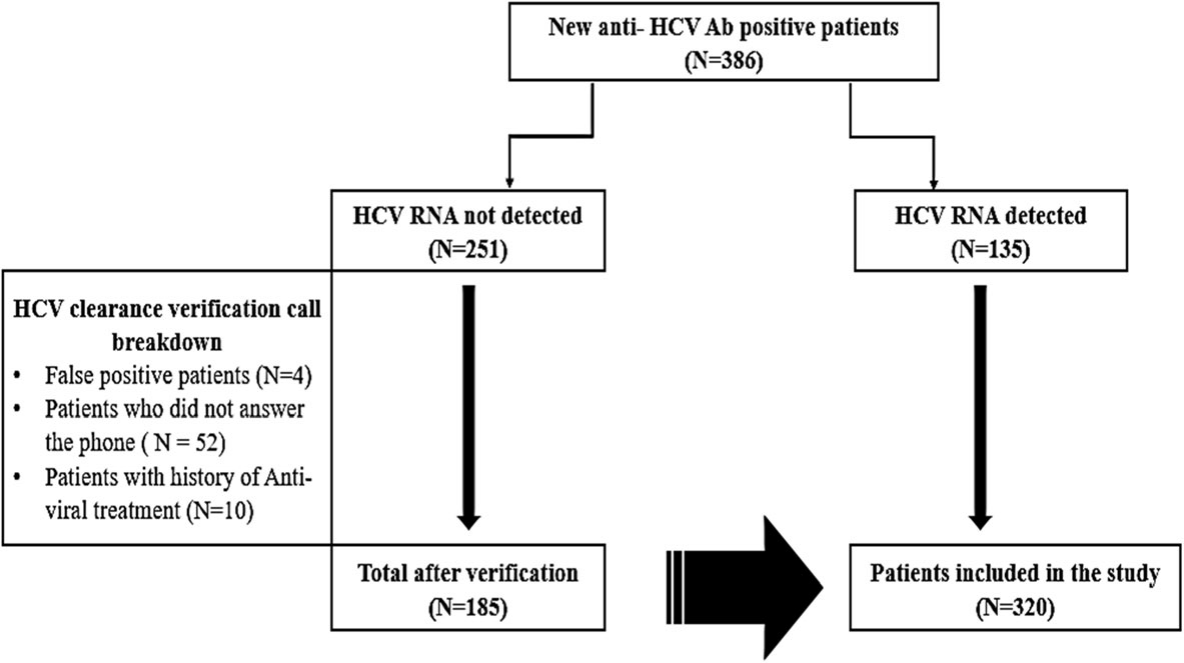
HCV viral clearance population breakdown

Three hundred and eighty-six patients were considered newly identified HCV Ab Positive. Among this group, 135 patients had detectable HCV RNA indicating current active infection. Of the 251 HCV antibody positive/HCV RNA undetectable patients, four had a false positive anti-HCV antibody test result, ten had a prior history of successful HCV treatment with clearance of HCV infection due to treatment (i.e., not spontaneous clearance), and 52 could not be reached for verification of their prior history of HCV treatment (Fig. 1). These 66 patients were excluded from the study resulting in a total of 320 study patients – 135 HCV antibody positive/HCV RNA positive (i.e., infection not cleared) and 185 HCV antibody positive/ HCV RNA negative (i.e., spontaneous clearance). The majority of the study population was born between 1945 and 1965 (62%) and 56% were male. Whites/Caucasians represented the single largest race/ethnicity group (64%), followed by Black/African American (13%) and Hispanic/Latino (9%) (Table 1). Among our study population of newly identified HCV antibody positive patients participating in a HCV screening program 58% spontaneously cleared HCV.

**Table 1:** Demographic and clinical characteristics of newly identified HCV antibody positive patients, UCLA Health system, November 2015–September 2017

	HCV Antibody Positive^a^ (*n* = 320)	
	*n*	%
Age group (years)		
18–52	99	31.4
52–72 (“Baby Boomers”)	198	61.5
≥ 73	23	7.1
Sex		
Male	179	55.9
Female	140	44.1
Race/Ethnicity		
Asian or Pacific Islander	5	1.8
Black or African American	38	13.4
Hispanic or Latino	25	8.8
Other^b^	33	11.6
White or Caucasian	183	64.4
*Clinical Characteristics*		
Hepatitis C RNA detected	135	42
HIV Positive	10	3.1
Chronic Kidney Disease^c^	23	7.2
Diabetes Mellitus	45	14.2
*Laboratory Results* ^d^		
ALT (U/L)	25 (17–47)	
AST(U/L)	26 (20–39)	
Platelet count	232 (188–271)	
Bilirubin (mg/dL)	0.5 (0.3–0.7)	

*Abbreviations*: *ALT* Alanine Transaminase, *AST* Aspartate Transaminase

^a^Sum may not equal total because of missing information

^b^includes participants self-identifying as multiracial

^c^CKD based on ICD-10 codes

^d^data presented as median and interquartile range

### Prevalence of HCV clearance

The prevalence of HCV clearance varied by a number of demographic and clinical characteristics with HCV clearance being somewhat higher among women (63% vs. 53%; *p* value = 0.07), although not meeting statistical significance, and varying by race/ethnicity (Table 2). Specifically, we note that HCV clearance was lowest among those who identified as Black/African American (37%).

**Table 2:** Prevalence and factors associated with absence of HCV RNA among newly identified HCV antibody positive patients, UCLA Health system, November 2015–2017

	HCV RNA negative		*P* value	Unadjusted OR (95% Cl)	Adjusted OR (95% CI)
	*n*	%			
Total	185	58.0	–	–	–
*Age group (years)*			0.17		
18–52	58	58.6		Ref	Ref
52–72 (“Baby Boomers”)	118	59.6		1.04 (0.64–1.70)	1.35 (0.75–2.39)
≥ 73	9	39.1		0.45 (0.18–1.15)	0.45 (0.16–1.30)
Sex			0.07		
Male	95	53.1		Ref	Ref
Female	88	63.3		1.53 (0.97–2.40)	1.53 (0.91–2.56)
Race/Ethnicity			0.02		
Asian	3	60.0		1.08 (0.18–6.59)	1.00 (0.14–5.44)
Black or African American	14	36.8		0.42 (0.21–0.86)	0.43 (0.21–0.92)
Hispanic or Latino	10	40.0		0.48 (0.20–1.12)	0.63 (0.26–1.57)
Other^a^	27	82.0		3.87 (1.43–10.51)	4.38 (1.54–12.44)
White or Caucasian	106	58.2		Ref	Ref
*Clinical Characteristics*					
HIV Positive			0.33		
Yes	4	40.0		0.48 (0.13–1.73)	–
No	180	58.3		Ref	
Chronic Kidney Disease^b^			0.06		
Yes	9	39.1		0.45 (0.19–1.06)	0.37 (0.14–1.03)
No	173	59.0		Ref	Ref
Diabetes Mellitus			0.76		
Yes	25	55.6		0.91 (0.48–1.71)	–
No	157	57.9		Ref	

*Abbreviations*: *OR* odds ratio, *CI* confidence interval

^a^includes participants self-identifying as multiracial

^b^CKD based on ICD-10 codes

### Factors associated with spontaneous HCV clearance

After adjusting for age and sex, we found that race/ethnicity was independently associated with spontaneous HCV clearance. Those who identified as African American/Black had a 57% decreased odds of HCV clearance as compared to Whites [adjusted odds ratio (aOR) =0.43; 95% confidence interval (CI) =0.21–0.92) (Table 2). Moreover, those diagnosed with chronic kidney disease (aOR = 0.34; 95% CI 0.14–1.03) had a tendency to decreased likelihood of spontaneous HCV clearance.

## Discussion

We investigated the frequency of and factors associated with spontaneous HCV viral clearance among patients participating in a hepatitis C screening program at a large urban health system in Los Angeles. In this study, 58% of patients with newly identified infection had evidence of HCV viral clearance. It should be noted that we only included newly identified HCV Ab positive patients with prior testing and verified treatment history through both chart review and patient interviews, which may explain the difference compared to other published studies [1, 4, 30]. Among our patient population, spontaneous HCV viral clearance varied by race/ethnicity and was somewhat less likely to occur among those with chronic kidney disease (CKD).

Consistent with previous studies, clearance was greater among white patients compared to their black counterparts [1, 4]. Previous studies on genetic markers perhaps could explain the increased proportion of spontaneous viral clearance among non-black patients [10, 32–34]. Key modification with natural killer (NK) cells populations, HLA class II alleles and IL28B polymorphism have been suggested to predict the relationship between ethnic characteristics and HCV clearance [1, 32, 35–37]. A study conducted by Golden-Mason et al. proposed that the proportion of NKp46 expression was lower among African Americans compared to their white counterparts [1, 35]. NK cells are known to be the immune system’s first line of defense against viral pathogens [38]. NK cells do this work by eliminating virus-infected cells directly via cytolytic mechanisms or indirectly by secreting cytokines [38, 39]. Moreover, HLA type II alleles have been suggested to have some conflicting roles however, research suggests their role with HCV clearance includes the fact that African Americans have weaker HCV specific immunity [37]. Furthermore, IL28B polymorphism is known to be the strongest host gene predictor of HCV clearance. Previous studies discuss that IL28B allele rs12979860 were less likely to be observed among persons of African descent compared to European descent. Study by Ge et al., outlines the fact that HCV clearance occurred in 36.4% among non-blacks compared to 9.3% among black persons [10]. The suggested mechanism along with previous studies provides some evidence that NK cells and other genetic markers can play a protective role in patients exposed to hepatitis C. However, despite studies on predictors of HCV clearance, the association between ethnic characteristics and HCV viral clearance is not well understood and should be explored further.

Our study also found that patients with chronic kidney disease were somewhat less likely to clear HCV infection compared to those without chronic kidney disease. Due to several clinical implications, in 2017, the Kidney Disease Improving Global Outcome organization drafted specific guidelines to increase HCV screening among chronic kidney disease patients [40]. Earlier studies propose that HCV infection may influence the development of chronic kidney disease by stimulating a series of immune reactions that targets the kidney, which ultimately leads to glomerulonephritis [29, 41]. According to Azmi et al., the mechanism related to glomerulonephritis and HCV infection is known to be immune-complex mediated. Researchers believe that deposition of the immune complexes can trigger glomerulonephritis [41]. Overall, we also observed that HCV was associated with chronic kidney disease, although from our cross-sectional study we cannot determine causation.

There are a number of limitations in our study including: (1) our modest sample size limits our ability for extensive analyses; (2) given the lack of information on the precise timing of HCV exposure and HCV infection we are unable to determine time to HCV clearance; (3) exclusion of patients whose HCV clearance status could not be verified could have biased our estimates of HCV clearance; (4) lack of information on viral subtype limited our ability to determine whether HCV subtype is associated with clearance; and (5) we included only “newly identified cases” of HCV, excluding patients with previous HCV positive results, thus our results are not generalizable to other populations nor do they represent an unbiased population estimate of HCV clearance. Finally, we were unable to verify a specific Race/Ethnicity for patients included in the “Other” category during data extraction, which could have potentially affected our conclusions on the associations between HCV clearance and Race/ethnicity. Despite those limitations, this is one of a few studies exploring difference in HCV spontaneous clearance among a screening population, with rigorous strategies employed to exclude those with previous treatment history or false positive testing status.

Future studies should explore on the social regulation of gene expression. According to Cole et al., social regulation of gene expression is a conceptual relationship between genes and social world [42]. Recent studies suggest clear associations between social factors and the regulations of the human genome [42–44]. Perhaps the decreased expression of NKp46 or other genetic factors observed among black patients can be attributed to their exposure to poor environmental, social and economic conditions. Asking and attempting to answer those questions might enable a better understanding of what drives genetic differences at the molecular level.

## Conclusions

Overall, this study confirms a higher frequency of HCV spontaneous clearance. In addition, this study identified certain subgroups more likely to clear HCV infection. Individuals who are at greater risk for HCV infection such as those who identify as African American and patients with chronic kidney disease should be prioritized for HCV screening and treatment initiatives.

## Data Availability

All data generated or analyzed during this study are available from the principal investigator on reasonable request.

## Abbreviations

ALT: Alanine Aminotransferase
AST: Aspartate Aminotransferase
CKD: chronic kidney disease
HCV: Hepatitis C
HIV: Human Immunodeficiency Virus
ICD-10: International classification of diseases
UCLA: University of California, Los Angeles
